# The associations between working conditions and subjective sleep quality among female migrant care workers

**DOI:** 10.3389/fpubh.2023.1094513

**Published:** 2023-04-14

**Authors:** I-Ming Chen, Tzu-Yun Lin, Yi-Ling Chien, Jennifer Yi-Ying Chen, Jen-Hui Chan, Shih-Cheng Liao, Po-Hsiu Kuo, Hsi-Chung Chen

**Affiliations:** ^1^Department of Psychiatry, National Taiwan University Hospital, Taipei, Taiwan; ^2^Department of Psychiatry, National Taiwan University Medical College, Taipei, Taiwan; ^3^Department of Psychiatry, Far Eastern Memorial Hospital, New Taipei City, Taiwan; ^4^Department of Psychiatry, National Taiwan University Hospital Hsin-Chu Branch, Hsin-Chu, Taiwan; ^5^Department of Public Health, Institute of Epidemiology and Preventive Medicine, National Taiwan University, Taipei, Taiwan; ^6^Center of Sleep Disorders, National Taiwan University Hospital, Taipei, Taiwan

**Keywords:** migrant, care worker, sleep quality, working conditions, minority

## Abstract

**Background:**

Subjective sleep quality may reflect the mental well-being of migrant care workers; however, the related occupational factors remain unclear. This study examines the association between the characteristics of care labor and the subjective sleep quality of female migrants.

**Methods:**

In this cross-sectional study, Southeast Asian migrant care workers in Taiwan were recruited using convenience sampling. Data on working conditions, including workplace setting, wage, working hours, psychiatric symptoms of care recipients, and sleep quality measured using the Pittsburgh Sleep Quality Index (PSQI), were collected through computer-assisted personal interviews. Multiple linear regression analyses were performed to determine the independent relationship between working conditions and the PSQI global score.

**Results:**

There were 220 institution-(47.7%) and home-based (52.3%) care workers, and 47.7% had a PSQI score higher than 5. After controlling for covariates, the lowest tertile of wages and daily working hours (> 8 h) were independently correlated with poor sleep quality. Moreover, in the stepwise regression model, wage and working hours remained the most explainable correlates of poor sleep quality.

**Conclusion:**

This study lent support to the notion that low wages and long working hours are significant occupational factors that negatively impact the subjective sleep quality of female Southeast Asian migrant care workers in Taiwan.

## 1. Introduction

Demand for eldercare is expanding in many post-industrial countries (1). However, the long-term care sector has been haunted by labor shortages due to persistent structural problems, including understaffing, job insecurity, and complex skill demands without sufficient training (1). The unsatisfactory compensation, arduous workload, and devalued social position, resulted in difficult recruitment and low retention rate of long-term care workers; most modern societies rely on minorities, such as female migrants, to sustain the need for long-term care in their aging populations (2–4).

However, although a wide gap existed in the working conditions between migrants and native care workers (5, 6), the mental health consequences for these migrant care workers were rarely acknowledged by academic work. The lack of conclusive evidence regarding mental well-being among migrant care workers may be due to inadequate research methodologies. Although in-depth interviews have revealed that migrant care workers constantly feel tense at work (4, 7), quantitative studies have shown no or only mild psychological stress (6, 8). These results might underestimate the mental distress among migrant care workers, since they might be reluctant to disclose their psychological suffering for fear of employer retaliation or risk of deportation (7). Moreover, conventional in-person surveys conducted among immigrant populations tended to be biased by social desirability and language barriers (9). To overcome the limitations of conventional survey methods, the use of computer-assisted personal interviews (CAPI) could improve the reliability of survey results. Such survey methods could also protect privacy and increase participant autonomy for sensitive issues, such as mental disorders, sexual behaviors, and HIV infection (10–12). To the best of our knowledge, no study has utilized CAPI to facilitate mental health research in migrant care workers.

Instead of methodological improvements, some studies used sleep duration as a proxy for the mental well-being of migrant care workers, which was less confidential than other psychiatric measurements (8, 9, 13). Nonetheless, some migrant care workers reported unsatisfactory sleep quality despite having a subjective normal length of sleep (8). It was challenging to accurately assess the total length of their fragmented sleep, especially for home-based care workers who provide round-the-clock services. In contrast, self-reported sleep quality had a higher predictive power than sleep quantity for physical and psychiatric comorbidities (14–16). Subjective sleep quality also mediates a larger proportion of the relationship between structural adversities and health than does sleep duration (17). Therefore, sleep quality may be more informative than sleep duration in the psychosocial context of migrant care workers.

Jackson et al. (18) examined racial disparities in sleep among various kinds of occupations and suggested that structural inequities in the workplace influenced sleep between immigrants and native workers. In the healthcare industry, low socioeconomic status, long working hours, and frequent night shifts were predictors of differences in sleep duration between African or Caribbean immigrants and Native White employees (19). These studies revealed that occupational factors played a pivotal role in the racial disparity in sleep, and that understanding occupational determinants of sleep could be imperative to promote the mental health of migrant care workers. Nevertheless, the exact work-related factors that affect subjective sleep quality remain unclear.

In this study, we recruited Southeast Asian female migrant care workers in Taiwan and collected data using mobile-based in-person surveys. This study aimed to investigate the correlation between working conditions and subjective sleep quality. We hypothesized that disadvantaged working conditions, including low wages, extended work hours, and increased psychiatric symptoms in care recipients, would be associated with poor subjective sleep quality among migrant care workers.

## 2. Methods

### 2.1. Study participants

The global care workers’ labor market is highly gender-segregated and female-predominant (20). There were 219,295 migrant workers in the social welfare sector of Taiwan in May 2022, but over 99% of them were female from nearby Southeast Asian countries (21). Therefore, only females were recruited in this cross-sectional study. Migrant care workers who were above 18 years of age, whose country of origin was Indonesia, Vietnam, or the Philippines, and who had been working in Taiwan as in-home care workers or employees of nursing or residential institutions were eligible. In addition, the participants required English or Chinese fluency and the ability to read questionnaires written in English, Chinese, Indonesian, or Vietnamese. Eligible participants were recruited using convenience sampling between January 2021 and November 2021. All participants were introduced to the research assistants through the agency leaders and a non-profit organization for migrant workers. The study was conducted in accordance with the Helsinki Declaration of 1975, as revised in 2008. Written informed consent was obtained from all study participants. This study was approved by the institutional review board of the National Taiwan University Hospital (number 201912142RINB).

### 2.2. Data collection

Data were collected through CAPI, using the Qualtrics online survey platform. All the structured questionnaires used in this study validated the Indonesian and Vietnamese versions. We commissioned two professional translation services to translate the remaining survey content into Indonesian and Vietnamese and asked both parties to reconcile any translation inconsistencies to reach a consensus. This version was then read by Indonesian and Vietnamese people from a non-profit organization who were familiar with both cultures and languages to ensure that the final version was semantically accurate.

The participants either used their own smartphones or tablet computers provided by the researchers to complete the survey. During the interview process, participants were always able to contact nearby research assistants if they had any questions regarding the online questionnaires. Respondents who were interrupted by their duties could finish the survey later on that day during their available time since the Qualtrics platform allowed for temporary storage and asynchronous messaging.

### 2.3. Variables of working conditions

Based on previous studies targeting medical professionals and caregivers, factors contributing to poor sleep quality include wages (22), working hours (13), psychiatric symptoms of the care recipient (23–25), subjective stress from nursing work (23, 26), and depression (25). Therefore, in addition to basic sociodemographic data (age, sex, nationality, and educational attainment in years), our online survey inquired about participants’ psychiatric history (presence vs. absence); work conditions, including workplace setting (private residence vs. nursing institution); monthly wage (lowest tertile vs. upper and middle tertiles); and daily working hours (> 8 vs. ≤ 8). The literature suggests that the relationship between income and sleep quality could be non-linear (27, 28); thus, we defined the lowest tertile as the “case” for low income. In addition, psychiatric symptoms in care recipients, psychological distress, and subjective sleep quality were evaluated. Individuals were asked to report whether their care recipients had sleep disturbances; difficulties in communication; hallucinations or delusions; and hostility toward caregivers, namely, being verbally and/or physically aggressive. The respondents answered each question with either yes or no. The number of symptoms manifested was summed as a continuous variable.

### 2.4. Psychological distress

Participants completed the Patient Health Questionnare-9 (PHQ-9) in the version of their acquainted language to measure emotional distress. The PHQ-9 is a self-administered questionnaire that can be completed in approximately 3 min. It is used worldwide to screen for and assess depression severity. A higher PHQ-9 score indicates a higher severity of psychological distress within the past 2 weeks. The questionnaire is applicable in general primary care settings, demonstrating high reliability and validity, and is available in Chinese, English, Indonesian, and Vietnamese languages (29–32). The PHQ-9 consists of nine questions, of which the most significant ones can be used as references for the diagnosis of depression in the DSM-5. The summed score from the nine questions also reflects depression severity.

### 2.5. Self-reported sleep quality

We used the Pittsburgh Sleep Quality Index (PSQI) to assess the sleep quality of the study participants within the past month (33). The PSQI is a self-administered questionnaire that takes approximately 5–10 min to complete. It comprises 19 questions that assess seven domains pertaining to sleep: subjective sleep quality, sleep onset latency, total sleep duration, sleep efficiency, sleep disturbance, use of sleep medication, and daytime dysfunction. A lower PSQI score indicates better sleep quality in the past month. The PSQI has been translated into multiple languages, including Indonesian, Chinese, Vietnamese, and English, all of which have high reliability and validity (33–36). Using a PSQI total score > 5 to distinguish poor sleepers from good sleepers yielded satisfactory sensitivity and specificity. In the present study, total component scores were used to reflect the subject’s sleep quality.

### 2.6. Statistical analysis

Statistical analyses were performed using Microsoft Excel 2019 and SPSS version 17.0 (SPSS Inc., Chicago, IL, United States). Descriptive statistics are presented as the mean ± SD for continuous variables and *n* (%) for categorical variables. We conducted a Pearson correlation analysis to assess the correlations between continuous independent variables and the total PSQI score. The t-test and analysis of variance (ANOVA) were used for univariate analysis of categorical predictors. All predictors were then subjected to multiple regression analysis with forced entry and stepwise entry methods to examine their independent effects on PSQI. Results yielding a value of *p* < 0.05 were considered statistically significant.

## 3. Results

A total of 220 migrant care workers of Vietnamese (24.5%), Indonesian (63.2%), and Filipino (12.3%) nationalities participated in this study. Table 1 shows the main sociodemographic and employment conditions of the migrant workers. The average age of the participants was 36.3 ± 7.4 years (range = 21–68), with an average educational attainment of 11.2 (± 2.5) years. Five percent of participants had a psychiatric history. Of the participants, 47.7% worked at nursing institutions, while 52.3% worked as private family caregivers. Migrant caregivers had salaries that ranged between $16,000–25,000 NTD at the time of interview, with an average of $19,923.2 (± 2,901.9) NTD (= $667.8 ± 97.3 USD). The average wage of the lowest tertile was $16,972.2 NTD (± 438.0), whereas that of the middle and upper tertiles was $21,107.1 NTD (± 2,611.4). Migrant caregivers worked for 11.8 (± 4.5) hours per day, and 76.8% of them worked for over 8 h per day. Their care recipients had 2.1 (± 1.7) psychiatric symptoms in average. Overall, 64.5% of the participants considered their caregiving work to be highly stressful. In terms of mood- and sleep-related factors, the average PHQ-9 score was 3.9 (± 4.3). The average PSQI global score was 5.5 (± 3.3), with 47.7% of the participants (*n* = 105) scoring higher than 5.

**Table 1:** Sociodemographic and clinical characteristics of participants (*n* = 220).^*^

	Total (*n* = 220)
	*n* (%)
Age (years; mean, SD)	36.3 ± 7.4
Education (years; mean, SD)	11.2 ± 2.5
Nationality
Vietnam	54 (24.5)
Indonesia	139 (63.2)
Philippines	27 (12.3)
Psychiatric history
No	209 (95.0)
Yes	11 (5.0)
Workplace setting
Nursing institution	105 (47.7)
Home	115 (52.3)
Wage (thousand, NTD; mean, SD)
Upper two tertiles	21.1 (2.61)
Lowest tertile	17.0 (0.44)
Daily workhour
≤8	48 (21.8)
>8	169 (76.8)
Number of psychiatric symptoms of care recipient (mean, SD)	2.1 ± 1.7
Subjective stress from care work
No	78 (35.5)
Yes	142 (64.5)
Patient Health Questionnaire-9 (mean, SD)	3.9 ± 4.3
Pittsburgh Sleep Quality Index (mean, SD)	5.5 ± 3.3

* Numbers of subjects do not equal because of missing values in variables.

Table 2 shows the correlations between the PSQI total score and continuous independent variables. The global PSQI score was negatively correlated with age (*r* = −0.16, *p* < 0.05) but positively correlated with the PHQ-9 score (*r* = 0.61, *p* < 0.01). Education and the number of psychiatric symptoms in care recipients were not significantly associated with the PSQI score.

**Table 2:** Correlation matrix between age, education, number of psychiatric symptoms of care recipients, Patient Health Questionnaire, and Pittsburgh Sleep Quality Index.

		1	2	3	4	5
1	Age (years)	1				
2	Education (years)	−0.15*	1			
3	Number of psychiatric symptoms of care recipients	0.11	−0.01	1		
4	Patient Health Questionnaire-9	−0.27**	−0.04	0.22**	1	
5	Pittsburgh Sleep Quality Index	−0.16*	0.01	0.05	0.61**	1

^*^Value of *p* < 0.05, ^**^value of *p* < 0.01.

As presented in Table 3, univariate analyses of categorical predictors showed that psychiatric history and daily working hours were not associated with the PSQI scores. However, significant between-group differences in sleep quality were found based on nationality (*p* < 0.001), workplace setting (*p* = 0.002), wage (*p* = 0.004), and subjective stress from care work (*p* = 0.004).

**Table 3:** Univariate analyses for factors associated with Pittsburgh Sleep Quality Index.

	Pittsburgh Sleep Quality Index	
	mean ± SD	Value of *p* for *t* test/ANOVA
Nationality
Vietnam	3.91 ± 3.03	<0.001
Indonesia	6.13 ± 3.31	
Philippines	5.78 ± 3.03	
Psychiatric history
No	5.52 ± 3.33	0.67
Yes	6.00 ± 3.55	
Workplace
Nursing institution	4.81 ± 3.35	0.002
Home	6.21 ± 3.20	
Wage
Upper two tertiles	5.13 ± 3.26	0.004
Lowest tertile	6.58 ± 3.33	
Daily workhour
≤8	4.90 ± 3.33	0.12
>8	5.74 ± 3.32	
Subjective stress from care work
No	4.68 ± 3.49	0.004
Yes	6.02 ± 3.16	

In Table 4, the multiple linear regression analysis shows an independent relationship between various working conditions and subjective sleep quality. We examined the collinearity statistics for our multiple linear regression model and found that the range of Variance Inflation Factor was 1.05–2.91, indicating a low to moderate degree of collinearity among the independent variables. After controlling for covariates, the association between workplace and subjective stress from care work and higher PSQI scores vanished. By contrast, participants within the lowest tertile of wages had a higher PSQI than those in the other two tertiles [B(se) = 0.83(0.41), *p* = 0.046]. Working for more than 8 h per day proved to be associated with a higher PSQI compared to the counterpart [B(se) = 1.01(0.44), *p* = 0.02]. Stepwise regression analysis was used to identify the most explainable associates of working conditions. Low income, long working hours, and high PHQ-9 scores appear to be robust risk indicators for poor sleep quality.

**Table 4:** Multiple linear regression analyses for factors associated with Pittsburgh Sleep Quality Index.

	Pittsburgh Sleep Quality Index
	Full model				Stepwise		
	B (se)	95% CI	Value of *p*		B (se)	95% CI	Value of *p*
Age (years)	0.02 (0.03)	(−0.03, 0.08)	0.36				
Education (years)	−0.05 (0.09)	(−0.22, 0.13)	0.62				
Nationality
Indonesia vs. Vietnam	0.64 (0.63)	(−0.59, 1.87)	0.30				
Philippines vs. Vietnam	1.73 (0.75)	(0.25, 3.20)	0.02				
Psychiatric history
Yes vs. No	0.25 (0.82)	(−1.37, 1.87)	0.76				
Workplace
Home vs. Nursing institution	0.28 (0.50)	(−0.71, 1.28)	0.57				
Wage
Lowest tertile vs. Upper two tertiles	0.83 (0.41)	(0.02, 1.64)	0.046	2	1.05 (0.40)	(0.26, 1.83)	0.01
Daily workhour
> 8 vs. ≤ 8	1.01 (0.44)	(0.15, 1.88)	0.02	3	0.86 (0.43)	(0.02, 1.70)	0.046
Number of psychiatric symptoms of care recipient (mean, SD)	−0.20 (0.13)	(−0.46, 0.05)	0.12				
Subjective stress from care work
Yes vs. No	0.62 (0.43)	(−0.23, 1.47)	0.15				
Patient Health Questionnaire-9	0.46 (0.05)	(0.36, 0.55)	<0.001	1	0.46 (0.04)	(0.38, 0.54)	<0.001

## 4. Discussion

Using CAPI, the present study examined the determinants of working conditions for subjective sleep quality in female migrant care workers in Taiwan. The findings indicate a high prevalence of poor sleep quality among migrant care workers. After partialling out rigorous confounding effects, lower wages and longer working hours turned out to be independently associated with poor sleep quality among various working conditions. To the best of our knowledge, this is the first study to specifically elucidate the link between disadvantaged employment conditions and subjective sleep quality among migrant care workers. Moreover, CAPI substantially reduces information bias and promotes information accessibility, making this study stand out.

### 4.1. Computer-assisted personal interviews enhances accessibility and validity of information

Although abundant literature has focused on the mental health condition of migrant care workers or compared their mental distress with that of native workers, the results were mixed (4, 6–8, 37–39). Methodological difference was one of the main attributes, in which anticipated rejection of psychiatric disabilities, linguistic unproficiency, and time deficiency were major challenges for studies in migrant workers (40, 41). Specifically, migrant workers were afraid of mental health stigmas that would prevent them from keeping their jobs, and some employers would warn respondents to not damage their reputations (9). As a result, interviewers tended to respond in a positive way during traditional interviews to make the process more welcoming. In the present study, potential biases were minimized by using an online survey platform that allowed for anonymous login to protect the privacy of the participants’ responses and enabled temporary storage and offline use to save the precious time of migrant care workers. Another strength of CAPI lies in its language flexibility. For example, online questionnaires could include as many as 16 languages in a study conducted in a region of high ethnic diversity (11). Reduction of language barriers could facilitate genuine responses that reflect their mental well-being.

### 4.2. High prevalence of poor sleep quality in migrant care workers

Approximately half of the migrant care workers in our study reported poor sleep quality. Previous research found that the sleep duration of migrants was shorter than that of native workers in the healthcare industry (18, 19), but direct comparisons of subjective sleep quality between migrant and native workers are lacking. In Germany, Korea, and Taiwan, the prevalence of poor sleep quality evaluated by the PSQI ranged from 30.3 to 40.9% among working populations (42–44). Although convenience sampling might have affected the estimation of the prevalence of poor sleep quality in this study, we attempted to increase the diversity of our community samples by recruiting volunteers from different sources, such as nursing agencies and non-governmental organizations. Compared to the statistics for the general public, migrant care workers reported a relatively high proportion of poor sleep quality. It is possible that occupation had a negative influence on mental well-being, in addition to pre-existing disadvantages in ethnicity and sex.

### 4.3. Working conditions and sleep quality

While many studies have examined racial disparities in health and gender segregation in the labor market, only a few have investigated the occupational health of migrant care workers (6, 37, 38). These works confined their focus on some aspects of care labors, such as job precarity, job strain, and COVID-19 pandemic. In contrast, in this study, we considered a set of more comprehensive variables for working conditions. In addition to wages (22) and working hours (13), psychiatric symptoms of the care recipient (23–25) and subjective stress from nursing work (23, 26) were also specified in the regression models because they were established risk indicators for medical professionals as well as caregivers, whereby migrant care workers also featured. We found that workplace setting, wages, and subjective stress from care labor had significant crude associations with sleep quality, but only wages remained in the adjusted model. In contrast, working hours were associated with poor sleep quality until the adjustment for confounders. Furthermore, the results of stepwise linear regression also ascertained wage and working hours as the most explanatory variables for sleep quality in migrant care workers. Compared with the workplace and subjective stress, wages and working hours are more subject to labor law regulations. Accordingly, our findings demonstrate the imperativeness of including comprehensive indicators of working conditions when researchers intend to identify pivotal and modifiable factors that promote laborers’ health.

This study showed that lower income was associated with worse subjective sleep quality. This result was consistent with surveys conducted among the general public, in which living in poverty caused problematic sleep (45). There may be a bidirectional relationship between poverty and poor sleep quality. In our study, participants were in similar socioeconomic status; thus, theory of “social causation” is preferred to “social drift” as the mechanism that underlie our findings. Although migrant workers in Taiwan may already have higher earnings than they have in their home countries, the “relatively low” incomes, indicated as the lowest tertile among participants in this study, may remain to incur feeling of “relative deprivation.” Evidence that workers who perceived their wages as unfair would report higher sleep dissatisfaction also supports our contention (22). Whether reducing income inequality or elevating basic income would benefit the mental health of migrant care workers needs further research.

Three-quarters of the participants in this study worked more than 8 h per day. Working for extended hours with unstable work schedules is a global phenomenon among migrant care workers (46, 47). While non-shift workers have been found to have an elevated risk of insomnia with prolonged day time working hours (48), the situation could be worse for live-in care workers who must adapt to irregular split-schedule sleep (9, 46). Notably, in this study, working hours were found to exhibit an adverse impact on sleep quality only in the adjusted model. From a statistical perspective, the deleterious effect of extended working hours on sleep quality may be neutralized by protective confounders in unadjusted analyses. Further investigations are warranted to examine protective factors, such as sleep hygiene behaviors or sleep–wake schedules, in workers with long working hours.

### 4.4. Psychometric properties of the PSQI-defined sleep quality

Existing research on the sleep health of migrant care workers has mostly focused on sleep duration or objective measurement of sleep quality (18, 19), but little is known about their subjective sleep quality. While subjective and objective measures of sleep quality have only modest correlations, their implications for mental health also differ (49). Compared with objective estimates of sleep quality, the extent to which subjective sleep quality (i.e., PSQI-defined sleep quality) correlates with mental health is greater (49, 50). Thus, the construct potentially shared between the PSQI and mental distress contrasted the robust roles of low wage and long working hours as predictors for poor sleep quality under the strong explanatory power from depression.

### 4.5. Limitations

A few limitations of this study should be considered when interpreting our results. First, causal inferences were not possible in this cross-sectional study. However, the key determinants of working conditions for sleep quality identified in this study, wage and working hours, were less likely the consequences, but should have preceded sleep quality. Second, because the participants of this study included only women, the generalizability of the main findings to male migrant workers is limited. Additionally, our participants were migrants who worked in long-term nursing facilities and private residences. The majority of our in-home caregivers were recruited from a non-profit organization, the largest learning community for Southeast Asian migrant workers in Taiwan. These participants had joined classes aimed at empowering migrant workers through practical knowledge and skills that they might actually represent the healthier subgroup of in-home care workers. This selection bias may mask the disadvantages of in-home work settings. Finally, the PHQ-9 was used to control for confounding effects of depression. However, one of the items in the PHQ-9 that measured night insomnia symptoms and sleep duration was similar to some measures in the PSQI. Thus, specifying the PHQ-9 in statistical models may incur concern for over-adjustment. However, subjective sleep quality is a global impression of overall sleep–wake health, which indicates a higher scope and different dimensions when compared to night insomnia symptoms and sleep duration (49, 50). Even under these circumstances, lower wages and longer working hours remained risk indicators for poor sleep quality, which further highlighted the significance of these two indicators.

## 5. Conclusion

While migrant care workers are filling the gap in the healthcare workforce in many rapidly aging societies, it is mandatory to improve the working conditions for migrant laborers. According to our findings, the regularization of wages and working hours may be essential to enhance the mental well-being of migrant care workers. In the future, an in-depth exploration of factors that underlie the link between wages and working hours with subjective sleep quality with more representative individuals is warranted.

## Data availability statement

The raw data supporting the conclusions of this article will be made available by the authors, without undue reservation.

## Ethics statement

The studies involving human participants were reviewed and approved by the institutional review board of the National Taiwan University Hospital (number 201912142RINB). The patients/participants provided their written informed consent to participate in this study.

## Author contributions

I-MC and P-HK conceived the study. T-YL, Y-LC, J-HC, and S-CL assisted to refer the participants and gave opinions on the study design. H-CC analyzed the data. I-MC prepared the original draft. JC reviewed and edited the manuscript. H-CC critically revised the manuscript and owns primary responsibility for the final content. All authors contributed to the article and approved the submitted version.

## Funding

This study was supported by grants from the National Taiwan University Hospital (NTUH 108-N4441) and the Ministry of Science and Technology, Taiwan (MOST-109-2314-B-002-143; MOST-110-2314-B-002 -096 -MY3), which had no role in study design, data collection and analysis, decision to publish, or preparation of the manuscript.

## Conflict of interest

The authors declare that the research was conducted in the absence of any commercial or financial relationships that could be construed as a potential conflict of interest.

## Publisher’s note

All claims expressed in this article are solely those of the authors and do not necessarily represent those of their affiliated organizations, or those of the publisher, the editors and the reviewers. Any product that may be evaluated in this article, or claim that may be made by its manufacturer, is not guaranteed or endorsed by the publisher.

## Abbreviations

CAPI: Computer-Assisted Personal Interviews
PHQ-9: Participants completed the Patient Health Questionnare-9
PSQI: Pittsburgh Sleep Quality Index
